# Supplementary material for: HHV-6B detection and host gene expression implicate HHV-6B as pulmonary pathogen after hematopoietic cell transplant
Source: Nat Commun. 2024 Jan 16;15:542. doi: 10.1038/s41467-024-44828-9 (PMC10791683; doi:10.1038/s41467-024-44828-9)
Supplement: Supplementary file 1 — Supplementary Information [file 41467_2024_44828_MOESM1_ESM.pdf]

SUPPLEMENTAL TABLES AND FIGURES

**Table S1. Etiologies of lower respiratory tract disease**	
**Etiology**	**Number of Events**
**Bacterial LRTD (N = 10 events in 10 participants)**	
Nocardia	1
Aspiration pneumonia and or VAP	4
Legionella spp.	3
Pseudomonas aeruginosa	1
MRSA	1
**Viral LRTD (N = 20 events in 18 participants)**	
Rhinovirus/enterovirus	5
Metapneumovirus	4
Coronavirus	2
Adenovirus	2
PIV3	2
RSV	1
Influenza A	1
**Multiple viruses**	**3**
CMV, rhinovirus, adenovirus	1
Influenza A and adenovirus	1
CMV, rhinovirus	1
**Fungal LRTD (N = 27 events in 26 participants)**	
Probable pulmonary aspergillosis	17
Proven pulmonary aspergillosis	1
Probable pulmonary mucormycosis	2
Possible invasive fungal pneumonia	7
**Idiopathic pneumonia syndrome (IPS) (N = 22 events in 18 participants)**	
With diffuse alveolar hemorrhage (DAH)	9
Without DAH	13
**Other (N = 11 events in 11 participants)**	
Negative microbiologic workup, not consistent with definition of IPS	11
**Multifactorial (N = 35 events in 30 participants)**	
**Bacterial and viral**	**13**
Pseudomonas spp. and rhinovirus	1
Bacterial pneumonia NOS and PIV4	1
Mycobacterium abscessus and metapneumovirus	1
E. coli and coronavirus	1
Streptococcus viridans, rhinovirus, and adenovirus	1
Bacterial pneumonia NOS and HHV-6 detected clinically	1
Klebsiella pneumoniae, *Nocardia spp.*, and PIV1	1
MSSA, rhinovirus	1
Haemophilus influenzae, rhinovirus	1
Streptococcus viridans and PIV3	1
Pseudomonas spp, adenovirus, rhinovirus	1
Bacterial pneumonia (aspiration), metapneumovirus	1
Bacterial pneumonia (aspiration), adenovirus	1
**Bacterial and fungal**	**9**
E. coli and probable pulmonary aspergillosis	2
Polymicrobial bacterial pneumonia and probable pulmonary aspergillosis	3
Pseudomonas spp. and probable pulmonary aspergillosis	1
Bacterial pneumonia (aspiration) and probable pulmonary aspergillosis	1

MSSA and possible fungal pneumonia	1
Bacterial pneumonia (aspiration) and possible fungal pneumonia	1
**Viral and fungal**	**4**
PIV3 and probable pulmonary aspergillosis	1
Metapneumovirus and probable pulmonary aspergillosis	1
Rhinovirus and probable pulmonary mucormycosis	1
Influenza B and possible pulmonary aspergillosis	1
**Bacterial, viral, and fungal**	**3**
Polymicrobial bacterial pneumonia, coronavirus, proven pulmonary aspergillosis	1
Stenotrophomonas maltophilia, rhinovirus, and probable pulmonary aspergillosis	1
Bacterial pneumonia (aspiration), PIV3, and probable pulmonary aspergillosis	1
**Possible infection and other non-infectious processes**	6

Table S2. HHV-6B-specific mRNA transcripts detected in BALF and plasma

**HHV-6B DNA+ BAL at any level**[a]	**HHV-6B *U38* mRNA only detected**	**HHV-6B *U90* mRNA only detected**	***U38* and *U90* both detected**	**No HHV-6B mRNA detected**
45	6 (13%)	6 (13%)	10 (22%)	23 (51%)
**HHV-6B DNA+ Plasma**				
18[b]	4[c] (22%)	2 (11%)	10 (56%)	2 (11%)

Data are presented as number (percentage).
[a]Only samples with HHV-6B DNA detected were tested for HHV-6B mRNA.
[b]19 participants had HHV-6B DNA in plasma samples, but 1 did not have an available PAXgene blood tube collected for testing.
[c]2 of these samples did not undergo testing for U90.

**Table S3. Receive operator characteristic curve analysis to determine the performance characteristics of a given HHV-6B DNA viral load threshold in the BAL for predicting the detection of HHV-6B-specific mRNA transcripts**											
**Viral load**	**Log10(viral load)**	**Estimated Probability**	**No. of Correctly Predicted Events**	**No. of Correctly Predicted Nonevents**	**No. of Nonevents Predicted as Events**	**No. of Events Predicted as Nonevents**	**Sensitivity**	**1 - Specificity**	**PPV**	**NPV**	**Specificity**
19.99	1.30091	.15836	20	71	22	0	1.00	.23656	.47619	1.00000	.76344
23.83	1.37704	.15837	20	72	21	0	1.00	.22581	.48780	1.00000	.77419
38.69	1.58760	.15841	20	73	20	0	1.00	.21505	.50000	1.00000	.78495
59.00	1.77085	.15847	20	74	19	0	1.00	.20430	.51282	1.00000	.79570
62.57	1.79638	.15848	19	74	19	1	.95	.20430	.50000	.98667	.79570
67.00	1.82607	.15849	19	75	18	1	.95	.19355	.51351	.98684	.80645
73.72	1.86761	.15851	18	75	18	2	.90	.19355	.50000	.97403	.80645
85.00	1.92942	.15854	18	76	17	2	.90	.18280	.51429	.97436	.81720
100.78	2.00337	.15859	18	77	16	2	.90	.17204	.52941	.97468	.82796
102.00	2.00860	.15859	18	78	15	2	.90	.16129	.54545	.97500	.83871
111.00	2.04532	.15862	17	78	15	3	.85	.16129	.53125	.96296	.83871
141.72	2.15144	.15870	17	79	14	3	.85	.15054	.54839	.96341	.84946
145.00	2.16137	.15871	17	80	13	3	.85	.13978	.56667	.96386	.86022
179.43	2.25388	.15881	17	81	12	3	.85	.12903	.58621	.96429	.87097
218.39	2.33923	.15892	17	82	11	3	.85	.11828	.60714	.96471	.88172
227.00	2.35603	.15894	16	82	11	4	.80	.11828	.59259	.95349	.88172
231.00	2.36361	.15895	16	83	10	4	.80	.10753	.61538	.95402	.89247
253.77	2.40443	.15901	16	84	9	4	.80	.09677	.64000	.95455	.90323
281.00	2.44871	.15909	15	84	9	5	.75	.09677	.62500	.94382	.90323
284.00	2.45332	.15910	15	85	8	5	.75	.08602	.65217	.94444	.91398
392.53	2.59387	.15940	14	85	8	6	.70	.08602	.63636	.93407	.91398
407.72	2.61036	.15945	14	86	7	6	.70	.07527	.66667	.93478	.92473
480.00	2.68124	.15965	14	87	6	6	.70	.06452	.70000	.93548	.93548
533.00	2.72673	.15980	13	87	6	7	.65	.06452	.68421	.92553	.93548
577.96	2.76190	.15992	12	87	6	8	.60	.06452	.66667	.91579	.93548
600.33	2.77839	.15999	11	87	6	9	.55	.06452	.64706	.90625	.93548

1232.00	3.09061	.16177	11	88	5	9	.55	.05376	.68750	.90722	.94624
1457.00	3.16346	.16241	10	88	5	10	.50	.05376	.66667	.89796	.94624
1592.36	3.20204	.16279	10	89	4	10	.50	.04301	.71429	.89899	.95699
1909.00	3.28081	.16370	10	90	3	10	.50	.03226	.76923	.90000	.96774
2012.00	3.30363	.16399	9	90	3	11	.45	.03226	.75000	.89109	.96774
2835.00	3.45255	.16637	8	90	3	12	.40	.03226	.72727	.88235	.96774
7506.16	3.87542	.18036	7	90	3	13	.35	.03226	.70000	.87379	.96774
8901.75	3.94948	.18472	6	90	3	14	.30	.03226	.66667	.86538	.96774
9650.00	3.98453	.18709	5	90	3	15	.25	.03226	.62500	.85714	.96774
10831.46	4.03469	.19088	4	90	3	16	.20	.03226	.57143	.84906	.96774
14320.23	4.15595	.20240	3	90	3	17	.15	.03226	.50000	.84112	.96774
29414.75	4.46857	.25814	3	91	2	17	.15	.02151	.60000	.84259	.97849
55816.00	4.74676	.37673	2	91	2	18	.10	.02151	.50000	.83486	.97849
62431.00	4.79540	.40973	2	92	1	18	.10	.01075	.66667	.83636	.98925
114339.00	5.05819	.67275	1	92	1	19	.05	.01075	.50000	.82883	.98925
278408.00	5.44468	.98452	1	93	0	19	.05	.00000	1.00000	.83036	1.00000

Table S4. Association of BAL HHV-6B DNA detection ≥218 copies/mL with overall mortality and death from respiratory failure by day 60 after the BAL in adjusted Cox models

	Overall mortality		Death from respiratory failure	
**Variable**	**Adjusted HR (95% CI)**	**P**	**Adjusted HR (95% CI)**	**P**
**Model 1**				
**Age (years)**				
<60	Reference		Reference	
≥60	1.50 (0.77-2.94)	0.24	1.26 (0.59-2.67)	0.55
**Maximum oxygen use pre-BAL**[a]				
≤2 L/minute	Reference		Reference	
>2 L/minute	3.27 (1.60-6.71)	0.001	2.76 (1.25-6.09)	0.01
**Maximum corticosteroid use pre-BAL**[b]				
<1 mg/kg/day	Reference		Reference	
≥1 mg/kg/day	1.66 (0.80-3.42)	0.17	0.95 (0.36-2.50)	0.92
**HHV-6B DNA in BAL, ≥218 copies/mL**				
No	Reference		Reference	
Yes	1.81 (0.89-3.67)	0.099	2.35 (1.08-5.11)	0.03
**Model 2**[c]				
**LRTD cause**				
Bacterial/viral/fungal/multiple	2.35 (0.31-18.0)	0.41	N/A	N/A
IPS	1.84 (0.21-16.4)	0.59		
Other	Reference	Reference		
**Maximum oxygen use pre-BAL**[a]				
≤2 L/minute	Reference		N/A	N/A
>2 L/minute	3.19 (1.51-6.73)	0.002		
**Maximum corticosteroid use pre-BAL**[b]				
<1 mg/kg/day	Reference		N/A	N/A
≥1 mg/kg/day	1.87 (0.87-4.01)	0.11		
**HHV-6B DNA in BAL, ≥218 copies/mL**				
No	Reference		N/A	N/A
Yes	1.78 (0.87-3.63)	0.11		
**Model 3**[c]				
**Age (years)**				
<60	Reference			
≥60	1.55 (0.79-3.06)	0.20	N/A	N/A
**Antiviral use pre-BAL**				
No	Reference		Reference	
Yes	2.24 (1.12-4.51)	0.023	2.93 (1.31-6.55)	0.009
**Maximum oxygen use pre-BAL**[a]				
≤2 L/minute	Reference		Reference	
>2 L/minute	2.96 (1.42-6.14)	0.004	2.48 (1.07-5.72)	0.03
**Maximum corticosteroid use pre-BAL**[b]				
<1 mg/kg/day	Reference		Reference	
≥1 mg/kg/day	1.38 (0.66-2.90)	0.39	0.82 (0.28-2.38)	0.71
**HHV-6B DNA in BAL, ≥218 copies/mL**				
No	Reference		Reference	
Yes	1.84 (0.90-3.75)	0.095	2.54 (1.12-5.74)	0.03

CI indicates confidence interval; HHV-6B, human herpesvirus 6B; BAL, bronchoalveolar lavage.
Results are from univariate and multivariate Cox regression models with two-sided P values. No adjustments were made for multiple comparisons. 113 participants included in the model. There were 35 events for overall mortality and 27 events for death from respiratory failure (i.e., pulmonary death).
[a]Within 24 hours preceding the BAL.
[b]Within 14 days pre-BAL, based on prednisone-equivalent dosing.
[c]In Model 2, the LRTD variable was not further separated to avoid overfitting due to the limited number of events; inclusion of the LRTD variable in the pulmonary death model could not be accommodated for this reason. In Model 3, age was not incorporated into the pulmonary death model to avoid overfitting.

Table S5. Association of BAL HHV-6B DNA detection ≥578 copies/mL with overall mortality and death from respiratory failure by day 60 after the BAL in adjusted Cox models

	Overall mortality		Death from respiratory failure	
**Variable**	**Adjusted HR (95% CI)**	**P**	**Adjusted HR (95% CI)**	**P**
**Model 1**				
**Age (years)**				
<60	Reference		Reference	
≥60	1.41 (0.72-2.77)	0.31	1.12 (0.52-2.46)	0.77
**Maximum oxygen use pre-BAL[a]**				
≤2 L/minute	Reference		Reference	
>2 L/minute	3.65 (1.77-7.54)	<0.001	3.15 (1.34-7.37)	0.008
**Maximum corticosteroid use pre-BAL[b]**				
<1 mg/kg/day	Reference		Reference	
≥1 mg/kg/day	1.89 (0.90-3.97)	0.09	1.01 (0.39-2.62)	0.99
**HHV-6B DNA in BAL, ≥578 copies/mL**				
No	Reference		Reference	
Yes	2.86 (1.24-6.60)	0.01	2.79 (1.11-7.03)	0.03
**Model 2[c]**				
**LRTD cause**				
Bacterial/viral/fungal/multiple	2.11 (0.27-16.3)	0.47	N/A	N/A
IPS	1.93 (0.22-17.1)	0.55		
Other	Reference	Reference		
**Maximum oxygen use pre-BAL[a]**				
≤2 L/minute	Reference		N/A	N/A
>2 L/minute	3.09 (1.46-6.57)	0.003		
**Maximum corticosteroid use pre-BAL[b]**				
<1 mg/kg/day	Reference		N/A	N/A
≥1 mg/kg/day	2.08 (0.96-4.50)	0.06		
**HHV-6B DNA in BAL, ≥578 copies/mL**				
No	Reference		N/A	N/A
Yes	3.16 (1.47-6.79)	0.003		
**Model 3**				
**Age (years)**				
<60	Reference			
≥60	1.66 (0.85-3.26)	0.14	N/A	N/A
**Antiviral use pre-BAL**				
No	Reference		Reference	
Yes	2.15 (1.08-4.29)	0.03	2.70 (1.23-5.94)	0.01
**Maximum oxygen use pre-BAL[a]**				
≤2 L/minute	Reference		Reference	
>2 L/minute	2.91 (1.41-6.03)	0.004	2.41 (1.04-5.54)	0.04
**Maximum corticosteroid use pre-BAL[b]**				
<1 mg/kg/day	Reference		Reference	
≥1 mg/kg/day	1.78 (0.83-3.85)	0.14	1.02 (0.35-3.01)	0.97
**HHV-6B DNA in BAL, ≥578 copies/mL**				
No	Reference		Reference	
Yes	3.40 (1.57-7.39)	0.002	3.70 (1.48-9.20)	0.005

CI indicates confidence interval; HHV-6B, human herpesvirus 6B; BAL, bronchoalveolar lavage.
Results are from univariate and multivariate Cox regression models with two-sided P values. No adjustments were made for multiple comparisons. 113 participants included in the model. There were 35 events for overall mortality and 27 events for death from respiratory failure (i.e., pulmonary death).
[a]Within 24 hours preceding the BAL.
[b]Within 14 days pre-BAL, based on prednisone-equivalent dosing.
[c]In Model 2, the LRTD variable was not further separated to avoid overfitting due to the limited number of events; inclusion of the LRTD variable in the pulmonary death model could not be accommodated for this reason.

Table S6. Demographic and clinical variables at the time of the BAL in the subgroup of 54 patients with whole blood RNASeq testing

	Negative HHV-6B DNA in BAL (N=37)	Positive HHV6-B DNA in BAL (any level) (N=16)[a]	Total (N=53[b])
**Age, years**			
21-40	10 (27%)	6 (38%)	16 (30%)
41-60	14 (38%)	5 (31%)	19 (36%)
>60	13 (35%)	5 (31%)	18 (34%)
**Female sex**	12 (32%)	6 (38%)	18 (34%)
**Race**			
Caucasian	26 (70%)	12 (75%)	38 (72%)
Non-Caucasian	10 (27%)	4 (25%)	14 (26%)
Unknown	1 (3%)	0 (0%)	1 (2%)
**Year of HCT**			
2015	3 (8%)	0 (0%)	3 (6%)
2016	8 (22%)	2 (13%)	10 (19%)
2017	13 (35%)	3 (19%)	16 (30%)
2018	9 (24%)	6 (38%)	15 (28%)
2019	4 (11%)	5 (31%)	9 (17%)
**CMV serostatus**			
D- and R-	6 (16%)	2 (13%)	8 (15%)
D+ or R+	31 (84%)	12 (75%)	43 (81%)
Missing	0 (0%)	2 (13%)	2 (4%)
	6 (16%)	2 (13%)	8 (15%)
**HCT comorbidity index Score[c]**			
0 (low)	4 (11%)	1 (6%)	5 (9%)
1-2 (intermediate)	13 (35%)	5 (31%)	18 (34%)
>=3 (high)	20 (54%)	10 (63%)	30 (57%)
**HLA and donor**			
Matched related	4 (11%)	3 (19%)	7 (13%)
Matched unrelated	14 (38%)	6 (38%)	20 (38%)
Mismatched related	7 (19%)	1 (6%)	8 (15%)
Mismatched unrelated	12 (32%)	6 (38%)	18 (34%)
**Cell source**			
Peripheral blood	28 (76%)	13 (81%)	41 (77%)
Bone marrow	7 (19%)	3 (19%)	10 (19%)
Umbilical cord blood	2 (5%)	0 (0%)	2 (4%)
**Myeloablative conditioning[d]**	18 (49%)	7 (44%)	25 (47%)

**Maximum corticosteroid use pre-BAL[e]**			
None	17 (46%)	6 (38%)	23 (43%)
<2 mg/kg/day	17 (46%)	9 (56%)	26 (49%)
≥2 mg/kg/day	3 (8%)	1 (6%)	4 (8%)
**Maximum oxygen use pre-BAL >2 L/minute[f]**	15 (41%)	8 (50%)	23 (43%)
**WBC count pre-BAL[g]**			
>1,000 cells/mm^3	33 (89%)	16 (100%)	49 (92%)
≤1,000 cells/mm^3	3 (8%)	0 (0%)	3 (6%)
Missing	1 (3%)	0 (0%)	1 (2%)
**ALC pre-BAL[g]**			
>300 cells/mm^3	19 (51%)	4 (25%)	23 (43%)
≤300 cells/mm^3	17 (46%)	11 (69%)	28 (53%)
Missing	1 (3%)	1 (6%)	2 (4%)
**ANC pre-BAL[g]**			
>500 cells/mm^3	32 (86%)	13 (81%)	45 (85%)
≤500 cells/mm^3	4 (11%)	2 (13%)	6 (11%)
Missing	1 (3%)	1 (6%)	2 (4%)
**Day of BAL post-HCT (median, IQR)**	43 (27-76)	44 (28-77)	43 (27-76)
**LRTD cause[h]**			
Bacterial	5 (14%)	2 (13%)	7 (13%)
Viral	11 (30%)	6 (38%)	17 (32%)
Fungal	11 (30%)	6 (38%)	17 (32%)
IPS	10 (27%)	2 (13%)	12 (23%)

Data are presented as number (percentage), unless otherwise indicated.
HHV-6B indicates human herpesvirus 6B; BAL, bronchoalveolar lavage; D, donor; R, recipient; HLA, human leukocyte antigen; WBC, white blood cell; ALC, absolute lymphocyte count; ANC, absolute neutrophil count; LRTD, lower respiratory tract disease; IPS, idiopathic pneumonia syndrome.
[a]10 participants in this group had BAL HHV-6B viral load ≥218 copies/mL.
[b]1 of the 54 participants in this subgroup did not have a BAL fluid sample available for testing and are excluded from this Table.
[c]Based on the HCT-comorbidity index.
[d]Myeloablative regimens included any regimen containing ≥800 cGY TBI, any regimen containing carmustine/etoposide/cytarabine/melphalan (BEAM), or any regimen containing busulfan/cyclophosphamide with or without antithymocyte globulin.
[e]Within 14 days pre-BAL, based on prednisone-equivalent dosing.
[f]Within 24 hours preceding the BAL.
[g]Closest sample within 3 days pre-BAL.

Table S7. Key characteristics in participants with blood genome-wide sequencing data stratified by LRTD category				
**IPS (N=12)**		**No HHV-6B ≥218 copies/mL in BALF or any detection in blood (N=8)**	**HHV-6B ≥218 copies/mL in BALF (with or without any blood detection) (N=2)**	**HHV-6B in blood (any level) but not detected in BALF (at ≥218 copies/mL) (N=2)**
Day post HCT	Median (IQR)	36 (25-59)	50 (23-76)	24 (19-29)
WBC[a]	Median (IQR)	3,800 (2,700-5,900)	3,690 (3,600-3,780)	15,670 (10,830-20,510)
ALC[a]	Median (IQR)	293 (132-371)	533 (227-839)	410 (0.0-820)
ANC[a]	Median (IQR)	3,120 (2,111-4,746)	2,582 (2,027-3,137)	10,226 (8,556-11,896)
Maximum corticosteroid use pre-BAL[b]	None	3 (38%)	2 (100%)	2 (100%)
	<2 mg/kg/day	4 (50%)	0	0
	≥2 mg/kg/day	1 (13%)	0	0
Receiving treatment for CMV or adenovirus with ganciclovir, foscarnet, or cidofovir at time of sample collection (N=4)		3 (38%)	0	1 (50%)
**Viral (N=17)**		**No HHV-6B ≥218 copies/mL in BALF or any detection in blood (N=13)**	**HHV-6B ≥218 copies/mL in BALF (with or without any blood detection) (N=3)**	**HHV-6B in blood (any level) but not detected in BALF (at ≥218 copies/mL) (N=1)**
Day post HCT	Median (IQR)	85 (56-92)	34 (27-57)	21 (21-21)
WBC[a]	Median (IQR)	4,790 (2,900-6,000)	6,280 (4,330-8,560)	2,740

ALC[a]	Median (IQR)	564 (203-684)	217 (0-342)	137
ANC[a]	Median (IQR)	3,308 (1,836-4,803)	5,401 (2,685-5,992)	1,754
Maximum corticosteroid use pre-BAL[b]	None	5 (38%)	2 (67%)	1 (100%)
	<2 mg/kg/day	7 (54%)	0	0
	>=2 mg/kg/day	1 (8%)	1 (33%)	0
Receiving treatment for CMV or adenovirus with ganciclovir, foscarnet, or cidofovir at time of sample collection (N=8)		7 (54%)	1 (33%)	0
**Fungal (N=17)**		**No HHV-6B ≥218 copies/mL in BALF or any detection in blood (N=13)**	**HHV-6B ≥218 copies/mL in BALF (with or without any blood detection) (N=4)**	**HHV-6B in blood (any level) but not detected in BALF (at ≥218 copies/mL) (N=0)**
Day post HCT	Median (IQR)	41 (15-76)	23 (16-30)	--
Total WBC[a]	Median (IQR)	3,020 (1,300-5,000)	3,535 (3,410-3,730)	--
ALC[a]	Median (IQR)	172 (86-360)	166 (110-319)	--
ANC[a]	Median (IQR)	1,685 (418-3,934)	2,514 (2,032-3,313)	--
Maximum corticosteroid use pre-BAL[b]	None	5 (38%)	3 (75%)	--
	<2 mg/kg/day	8 (62%)	1 (25%)	--
	>=2 mg/kg/day	0	0	--

Receiving treatment for CMV or adenovirus with ganciclovir, foscarnet, or cidofovir at time of sample collection (N=4)		4 (31%)	0	0
**Bacterial (N=7)**		**No HHV-6B ≥218 copies/mL in BALF or any detection in blood (N=6)**	**HHV-6B ≥218 copies/mL in BALF (with or without any blood detection) (N=1)**	**HHV-6B in blood (any level) but not detected in BALF (at ≥218 copies/mL) (N=0)**
Day post HCT	Median (IQR)	46 (36-83)	52	--
Total WBC[a]	Median (IQR)	7,000 (1,910-9,280)	8,800	--
ALC[a]	Median (IQR)	420 (91-516)	185	--
ANC[a]	Median (IQR)	6,020 (1,665-7,702)	216	--
Maximum corticosteroid use pre-BAL[b]	None	2 (33%)	0	--
	<2 mg/kg/day	3 (50%)	1 (100%)	--
	>=2 mg/kg/day	1 (17%)	0	--
Receiving treatment for CMV or adenovirus with ganciclovir, foscarnet, or cidofovir at time of sample collection (N=3)		3 (50%)	0	0

Data are presented as number (percentage), unless otherwise indicated. Percentages are column percentages.
HHV-6B indicates human herpesvirus 6B; BAL, bronchoalveolar lavage; WBC, white blood cell; ALC, absolute lymphocyte count; ANC, absolute neutrophil count; IPS, idiopathic pneumonia syndrome.
[a]Closest sample within 3 days pre-BAL. Units of cells/mm^3.
[b]Within 14 days pre-BAL, based on prednisone-equivalent dosing.

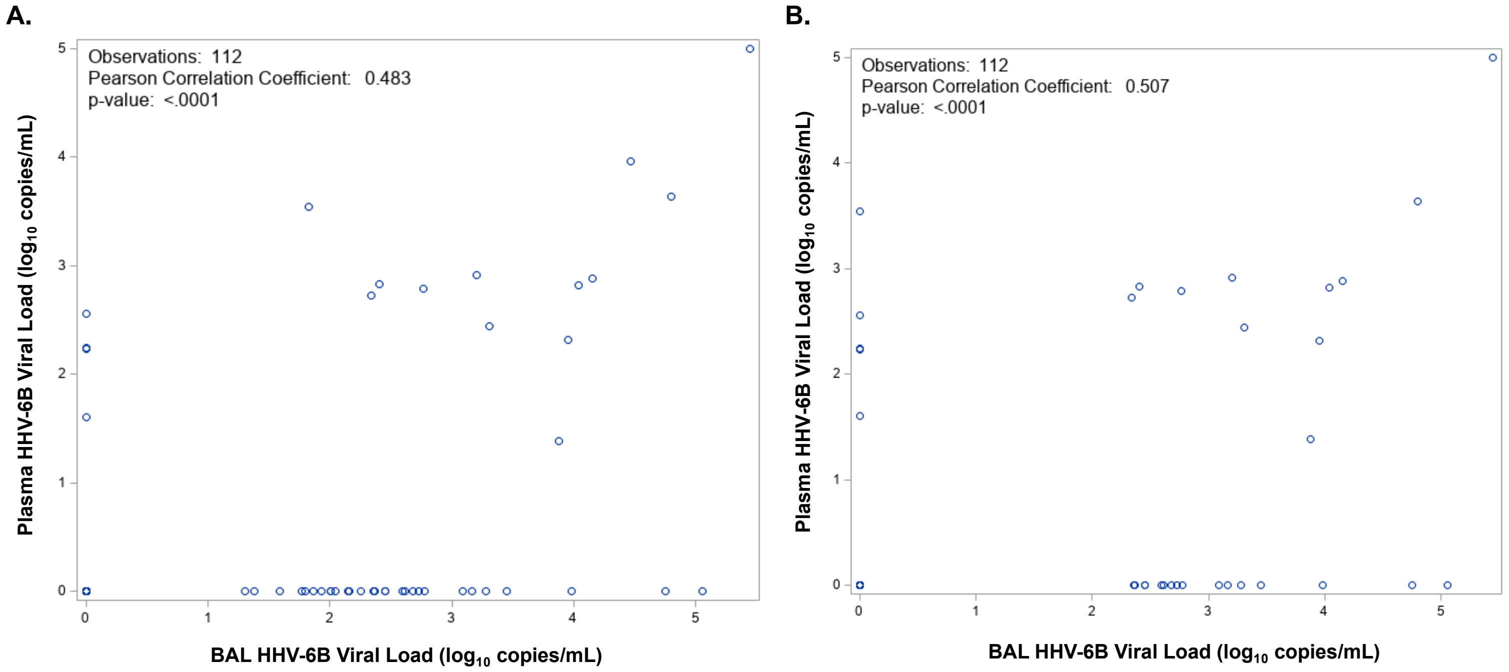

Figure S1. Correlation of BAL and plasma HHV-6B viral load.
Correlation of BAL and plasma HHV-6B viral load (**A**). With categorization of BAL fluid HHV-6B viral loads <218 copies/mL as 0 (i.e., indicative of viral shedding), there is higher correlation (**B**).

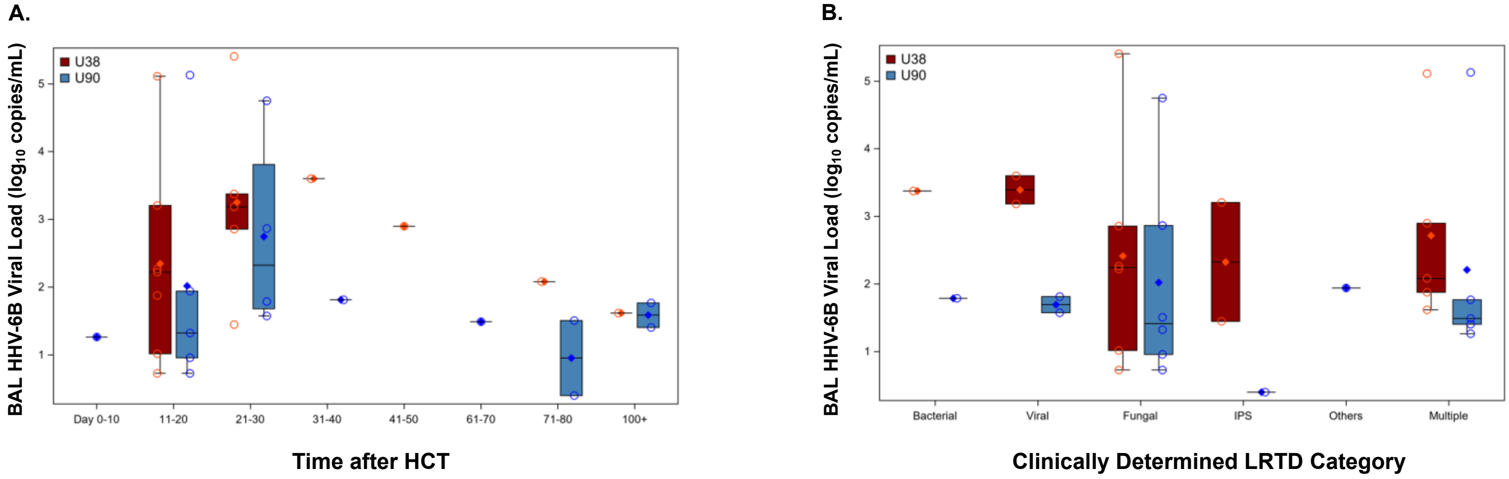

Figure S2. BAL fluid HHV-6B mRNA viral load over time and by LRTD category.
Data are shown from 22 BAL events that had HHV-6B mRNA detection for either the *U38* or *U90* transcript. HHV-6B mRNA detection in categories of time are shown in **Panel A**. HHV-6B mRNA detection in lower respiratory tract disease (LRTD) categories are shown in **Panel B**. The boxes represent the interquartile range, the horizontal lines and diamonds within the boxes represent the median and mean, respectively, and the upper and lower whiskers extend to the third and first quartiles plus or minus 1.5 times the interquartile range, respectively. Circles represent data points.

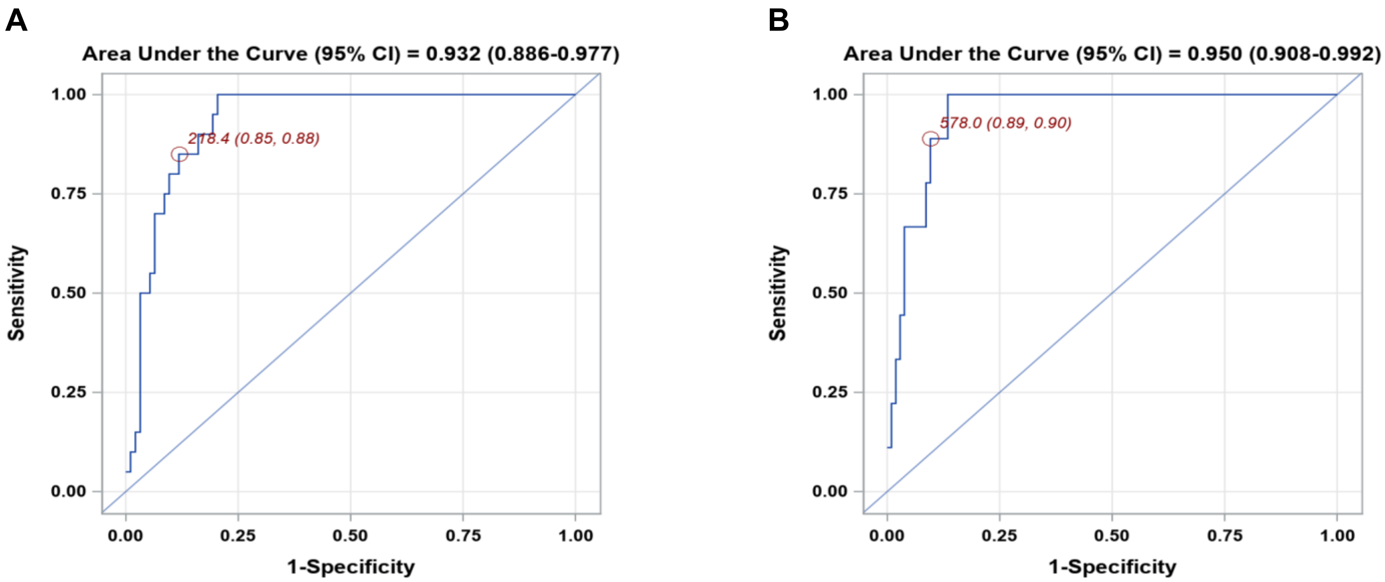

Figure S3. Receive operator characteristic (ROC) curve analyses to determine the performance characteristics of a given HHV-6B DNA viral load threshold in the BAL for predicting the detection of HHV-6B-specific mRNA transcripts (*U38* and/or *U90*).

A viral load of 2.3 $\log_{10}$ copies/mL (218 copies/mL) maximized sensitivity (85%) and specificity (88%) for the detection of either HHV-6B *U38* or *U90* mRNA **(A)**. Negative and positive predictive values are demonstrated in **Table S3**. An ROC curve for predicting the detection of both *U38* and *U90* in the same sample identified a viral load threshold of ≥2.8 $\log_{10}$ copies/mL (578 copies/mL) as the optimal cut point with AUC = 0.95 (95% CI, 0.91-0.99), sensitivity = 89%, and specificity = 90% **(B)**.

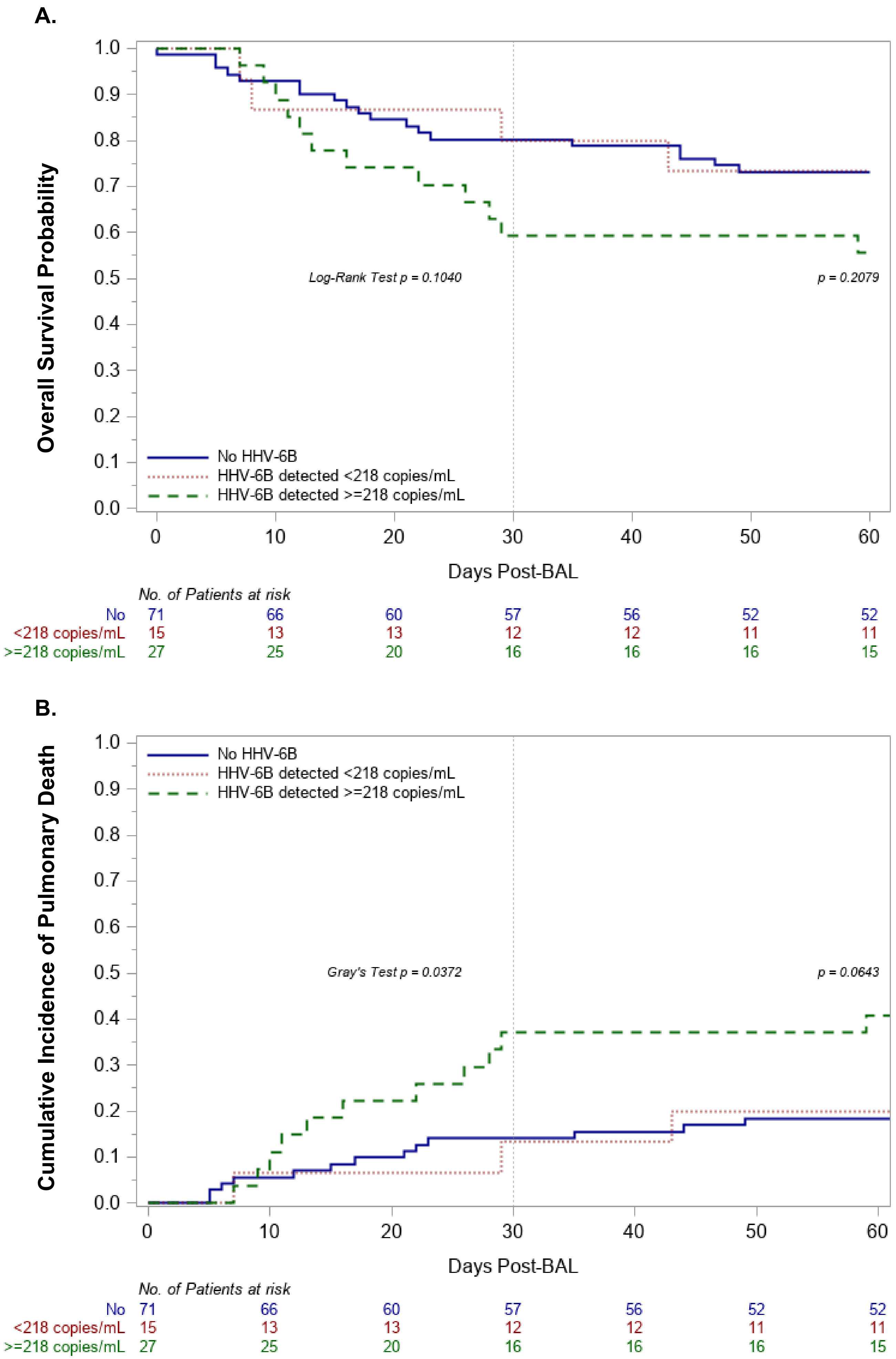
A.
Overall Survival Probability
1.0
0.9
0.8
0.7
0.6
0.5
0.4
0.3
0.2
0.1
0.0
Log-Rank Test p = 0.1040
p = 0.2079
No HHV-6B
HHV-6B detected <218 copies/mL
HHV-6B detected >=218 copies/mL
0
10
20
30
40
50
60
Days Post-BAL
No. of Patients at risk
No 71 66 60 57 56 52 52
<218 copies/mL 15 13 13 12 12 11 11
>=218 copies/mL 27 25 20 16 16 16 15
B.
Cumulative Incidence of Pulmonary Death
No HHV-6B
HHV-6B detected <218 copies/mL
HHV-6B detected >=218 copies/mL
Gray's Test p = 0.0372
p = 0.0643
Days Post-BAL
No. of Patients at risk
No 71 66 60 57 56 52 52
<218 copies/mL 15 13 13 12 12 11 11
>=218 copies/mL 27 25 20 16 16 16 15

Figure S4. Kaplan Meier and cumulative incidence plots of time to overall mortality and death due to respiratory failure, respectively, among patients with BAL fluid HHV-6B viral load ≥218 copies/mL, detected but <218 copies/mL, and not detected.

Participants with HHV-6B DNA ≥218 copies/ml (2.3 $\log_{10}$ copies/mL) in BALF based on the threshold predictive of mRNA detection (n=27) had increased overall mortality (adjusted hazard ratio [aHR], 1.81; 95% CI, 0.89-3.67; p=0.099) (**A**) and death from respiratory failure (aHR, 2.35; 95% CI, 1.08-5.11; p=0.032) (**B**) compared to those with HHV-6B detection but at levels <2.3 $\log_{10}$ copies/mL and those without HHV-6B detection in models adjusted for age, oxygen use, and steroid use at the time of BAL in a multivariable Cox regression model. Deaths due to other causes were considered competing events in the cumulative incidence curve. The first and second log-rank or Gray's test statistics in the figures depict the unadjusted comparison of the curves at day 30 and day 60 post-BAL, respectively.
