# Supplementary material for: HHV-6B detection and host gene expression implicate HHV-6B as pulmonary pathogen after hematopoietic cell transplant
Source: Nat Commun. 2024 Jan 16;15:542. doi: 10.1038/s41467-024-44828-9 (PMC10791683; doi:10.1038/s41467-024-44828-9)
Supplement: Supplementary file 3 — Reporting Summary [file 41467_2024_44828_MOESM3_ESM.pdf]

nature portfolio

Corresponding author(s): Hill, Joshua

Last updated by author(s): Dec 18, 2023

Reporting Summary

Nature Portfolio wishes to improve the reproducibility of the work that we publish. This form provides structure for consistency and transparency in reporting. For further information on Nature Portfolio policies, see our Editorial Policies and the Editorial Policy Checklist.

Statistics

For all statistical analyses, confirm that the following items are present in the figure legend, table legend, main text, or Methods section.

n/a	Confirmed	
☐	☒	The exact sample size (*n*) for each experimental group/condition, given as a discrete number and unit of measurement
☐	☒	A statement on whether measurements were taken from distinct samples or whether the same sample was measured repeatedly
☐	☒	The statistical test(s) used AND whether they are one- or two-sided<br>*Only common tests should be described solely by name; describe more complex techniques in the Methods section.*
☐	☒	A description of all covariates tested
☐	☒	A description of any assumptions or corrections, such as tests of normality and adjustment for multiple comparisons
☐	☒	A full description of the statistical parameters including central tendency (e.g. means) or other basic estimates (e.g. regression coefficient) AND variation (e.g. standard deviation) or associated estimates of uncertainty (e.g. confidence intervals)
☐	☒	For null hypothesis testing, the test statistic (e.g. *F*, *t*, *r*) with confidence intervals, effect sizes, degrees of freedom and *P* value noted<br>*Give P values as exact values whenever suitable.*
☒	☐	For Bayesian analysis, information on the choice of priors and Markov chain Monte Carlo settings
☒	☐	For hierarchical and complex designs, identification of the appropriate level for tests and full reporting of outcomes
☒	☐	Estimates of effect sizes (e.g. Cohen's *d*, Pearson's *r*), indicating how they were calculated

Our web collection on statistics for biologists contains articles on many of the points above.

Software and code

Policy information about availability of computer code

Data collection	No software was used.
Data analysis	Analyses of clinical and virologic data were conducted with SAS version 9.3 (SAS institute, Cary, NC). Principal Component Analysis was conducted with Partek Flow (Partek Inc., Chesterfield, MO, USA). We identified differentially expressed genes using the R (version 4.3) package DESeq2 (version 1.42). We applied Gene Set Enrichment Analysis (GSEA, version 4.3.1) using 50 Hallmark and 1329 Canonical Pathway gene sets curated from the Molecular Signature Database (MSigDB, version 2023.2; https://www.gsea-msigdb.org/gsea/index.jsp).

For manuscripts utilizing custom algorithms or software that are central to the research but not yet described in published literature, software must be made available to editors and reviewers. We strongly encourage code deposition in a community repository (e.g. GitHub). See the Nature Portfolio guidelines for submitting code & software for further information.

Data

Policy information about availability of data

All manuscripts must include a data availability statement. This statement should provide the following information, where applicable:

- Accession codes, unique identifiers, or web links for publicly available datasets
- A description of any restrictions on data availability
- For clinical datasets or third party data, please ensure that the statement adheres to our policy

The RNA-seq data generated in this study meet MINSEQE (Minimum Information About a Next-generation Sequencing Experiment) guidelines and have been

deposited in Gene Expression Omnibus under accession code GSE244210 (https://www.ncbi.nlm.nih.gov/geo/). Additional de-identified datasets generated and analysed for this study are available from the corresponding author (jahill3@fredhutch.org) after publication upon reasonable request, with investigator financial support, and with appropriate documentation of IRB approval and/or data access agreements as applicable. The authors will make best efforts to provide the requested information within three months of the request.

Research involving human participants, their data, or biological material

Policy information about studies with human participants or human data. See also policy information about sex, gender (identity/presentation), and sexual orientation and race, ethnicity and racism.

Reporting on sex and gender	Participant sex was abstracted from electronic medical records and is typically self-reported but was not solicited for this study. Gender was not collected. Participant sex was not considered in the study design but was evaluated in univariate models. Sex was collected in all 113 participants consisting of 49 (43%) women and 64 (56%) men.
Reporting on race, ethnicity, or other socially relevant groupings	Participant race was abstracted from electronic medical records and is typically self-reported but was not solicited for this study. Participant race was not considered in the study design but was evaluated in univariate models.
Population characteristics	We prospectively enrolled allogeneic HCT recipients undergoing a BAL for evaluation of lower respiratory tract disease within 120 days of HCT. The median age was 54.5 years old and ranged from 17 to 74.
Recruitment	Potential participants who met study eligibility criteria were identified by study teams through the electronic medical record or health care providers. Eligible participants were prospectively presented consent for this study. If an individual did not have capacity to consent, their legally authorized representative could consent on their behalf. Participants were not compensated. It is unlikely that this approach biased the study for or against the reported outcomes.
Ethics oversight	The study was approved by the Fred Hutchinson Cancer Center Institutional Review Board.

Note that full information on the approval of the study protocol must also be provided in the manuscript.

Field-specific reporting

Please select the one below that is the best fit for your research. If you are not sure, read the appropriate sections before making your selection.

☒ Life sciences ☐ Behavioural & social sciences ☐ Ecological, evolutionary & environmental sciences

For a reference copy of the document with all sections, see nature.com/documents/nr-reporting-summary-flat.pdf

Life sciences study design

All studies must disclose on these points even when the disclosure is negative.

Sample size	We performed a priori sample size calculations to estimate target sample sizes and power for detecting differences in overall mortality and gene expression based on preliminary data. We estimated that with a target sample size of approximately 150, we would identify up to 48 participants with any HHV-6B detection in BALF, about half of whom would have detectable HHV-6B mRNA transcripts (n=24). To test the hypothesis that patients with possible HHV-6B LRTD will have higher mortality than patients without, we estimated that these sample sizes would be able to demonstrate a statistically significant difference if the hazard ratio was at least 2.5-2.6 for effect sizes ranging from 5-20%, with 80% power and a two-sided type I error rate of 0.05. For the subgroup analyses with RNA-Seq, we estimated identifying 10-15 participants per LRTD category who met the described criteria. A power analysis for 2-group comparisons using the RNA-seq Power R package67 with parameters estimated from a representative RNA-seq dataset demonstrated that the study would be adequately powered (84%-95%) to detect meaningful changes in gene expression with ≥1.75-2 fold changes assuming a FDR of 5%.
Data exclusions	No data were excluded.
Replication	We did not have a validation cohort because of sample size limitations for this unique patient cohort and specimen biorepository.
Randomization	This was a prospective observational study, and randomization was not relevant to the design. Univariate and multivariate Cox proportional hazards models were used to compare clinical outcomes by day 60 after the BAL event.
Blinding	HHV-6 testing of samples was performed in batches after data were collected, so investigators were blinded to these results. Lab personnel were blinded to clinical data pertaining to the samples and participants.

Reporting for specific materials, systems and methods

We require information from authors about some types of materials, experimental systems and methods used in many studies. Here, indicate whether each material, system or method listed is relevant to your study. If you are not sure if a list item applies to your research, read the appropriate section before selecting a response.

Materials & experimental systems

n/a	Involved in the study
☒	☐ Antibodies
☒	☐ Eukaryotic cell lines
☒	☐ Palaeontology and archaeology
☒	☐ Animals and other organisms
☐	☒ Clinical data
☒	☐ Dual use research of concern
☒	☐ Plants

Methods

n/a	Involved in the study
☒	☐ ChIP-seq
☒	☐ Flow cytometry
☒	☐ MRI-based neuroimaging

Clinical data

Policy information about clinical studies

All manuscripts should comply with the ICMJE guidelines for publication of clinical research and a completed CONSORT checklist must be included with all submissions.

Clinical trial registration: Not applicable.

Study protocol: Not applicable (this was an observational cohort study).

Data collection: We prospectively enrolled adult allogeneic HCT recipients undergoing a BAL for evaluation of LRTD within 120 days of HCT from Fred Hutchinson Cancer Center (Fred Hutch), City of Hope Comprehensive Cancer Center (COH), and Memorial Sloan Kettering Cancer Center (MSKCC) from July 2015 through December 2019 . Data were abstracted from medical records for participant characteristics, microbiologic results, and outcomes for up to 60 days after the BAL date.

Outcomes: We generated Kaplan-Meier and cumulative incidence curves to estimate overall mortality and death due to respiratory causes (i.e., 'pulmonary death'), respectively, by 30 and 60 days after BAL in patients with and without possible HHV-6B pneumonitis, irrespective of other causes of LRTD. In the cumulative incidence curves, non-pulmonary deaths were competing events. Univariate and multivariate Cox proportional hazards models were used to compare these outcomes by day 60 after the BAL event, adjusted for potential confounders, including other causes of LRTD. Two-sided P values <0.05 were considered statistically significant.

Plants

Seed stocks: *Report on the source of all seed stocks or other plant material used. If applicable, state the seed stock centre and catalogue number. If plant specimens were collected from the field, describe the collection location, date and sampling procedures.*

Novel plant genotypes: *Describe the methods by which all novel plant genotypes were produced. This includes those generated by transgenic approaches, gene editing, chemical/radiation-based mutagenesis and hybridization. For transgenic lines, describe the transformation method, the number of independent lines analyzed and the generation upon which experiments were performed. For gene-edited lines, describe the editor used, the endogenous sequence targeted for editing, the targeting guide RNA sequence (if applicable) and how the editor was applied.*

Authentication: *Describe any authentication procedures for each seed stock used or novel genotype generated. Describe any experiments used to assess the effect of a mutation and, where applicable, how potential secondary effects (e.g. second site T-DNA insertions, mosiacism, off-target gene editing) were examined.*
